# A comparative study of localized phosphorus application and broadcasting method on biomass production and their use efficiency on Chilli (*Capsicum annuum*) under alkaline soil

**DOI:** 10.3389/fpls.2024.1304963

**Published:** 2024-03-01

**Authors:** Sanatan Kumar Swain, Sagolshem Kalidas-Singh, Anita Puyam, Dwipendra Thakuria, Soniya Maimom, R. Rustum Zhiipao, Ashim Debnath, Samikhya Bhuyan, Mayanglambam Homeshwari-Devi, Sangeeta Huidrom, Thupten Tsomu, Yashi Umbrey, Punam Bagang, Vikas Kumar Ravat, Bharati Lap, Avicha Tangjang, Devegowda S. R.

**Affiliations:** ^1^ School of Agriculture, Soil Science and Agricultural Chemistry, ITM University, Gwalior, MP, India; ^2^ Faculty of Agricultural Sciences, Rajiv Gandhi University, Doimukh, India; ^3^ College of Agriculture, Rani Lakshmi Bai Central Agricultural University, Jhansi, India; ^4^ Natural Resource Management, College of Post Graduate Studies in Agricultural Sciences, (Central Agricultural University-Imphal), Umiam, Meghalaya, India; ^5^ Department of Entomology, College of Horticulture, (Central Agricultural University-Imphal), Bermiok, South Sikkim, India; ^6^ All India National Project (AINP) Soil Biodiversity and Biofertilizer, Indian Council of Agricultural Research (ICAR)-Indian Institute of Soil Science, Bhopal, India; ^7^ Department of Pharmacology, Shri Guru Ram Rai Institute of Medical and Health Sciences, Dehradun, Uttarakhand, India

**Keywords:** rhizosphere based P application, P enrich slurry, root volume, biomass, P used efficiency

## Abstract

Rhizospheric based phosphorus (P) fertilizer management is necessary for crop production due to environmental concerns caused by the overuse of the broadcasting method and limited P reserves. This study proposes a comparison of P management that enhances P nutrition in Chilli (variety: *Arka Khyati*) through seedling root-dipping (SRD) in P-enriched slurry (SSP-amended; pH of 8.1), micro-dose placement (MDP; drill and place closer to plant root), and full dose (187.6 mg kg^-1^) placement by broadcasting (FD). In SRD, seedlings were dipped in five different P concentrations (50, 100, 200, 300, and 400 mg P_2_O_5_ kg^-1^) for varying durations (0, ½, 1, 2, 3, and 4 hours) and transplanted into pots (dipping in 0 mg P_2_O_5_ kg^-1^ consider as control), along with the MDP and FD treatments (total 33 treatments with 5 replications). [Seedlings dipped in 200, 300, and 400 mg P_2_O_5_ kg^-1^ died within a week after transplanting, thus were excluded from further analysis]. The amount of P received in MDP and FD were 21-90 times higher than P adhesion to seedling roots in SRD treatments. Root volume was in order SRD>MDP>FD. Seedlings dipped in 100 mg P_2_O_5_ kg^-1^ for 2 hours in SRD exhibited the highest biomass production, P-use and -recovery efficiency; and showed an increase of 52%, 178%, and 293% in FD, MDP, and SRD compared to the control respectively. It is recommended to use the SRD method with other P sources in reduced amount to maintain the native P pool in soil, and further multilocational trials are needed to validate.

## 1 Introduction

Phosphorus (P) is an essential macro-element for all living cells, but soil-grown plants are often deficient in it due to low concentrations in the soil solution and rapid immobilization and fixation of applied P (Hopkins and Hansen, 2019). Meanwhile, use of excessive P-fertilizer harms the environment and also challenges the food security in the scenario of depleting reserves rock phosphate crisis (Cordell et al., 2009). Recently, the cost of P fertilizers has increased after the Ukrainian-Russian war, creating additional challenges for developing countries to access and utilize these fertilizers (Brownlie et al., 2023). Under these circumstances, it is recommended that the management of P in agriculture should prioritize the reduction of P fertilizer usage (applied-P), investigation of potential recycling opportunities, and exploration of innovative rhizosphere-based methods of P application (Richardson et al., 2009).

In developing countries, it is a common practice to apply most of the P fertilizer through broadcasting or banded methods (Rahman and Zhang, 2018). However, there’s a drawback, that a considerable amount of the released soluble P quickly precipitates with other soil cations, turning into insoluble P complexes like Ca-P and Mg-P in alkaline soil, or Al-P and Fe-P in acidic soil. This makes the P unavailable for the plants. Therefore, in highly P-fixed soils, whether acidic or alkaline, P management should focus on rhizosphere based or localized P application approach (Kalidas-Singh and Thakuria, 2018; Goswami and Kalidas-Singh, 2023). Localized fertilizer application, which involves placing P fertilizer near to the seeds or seedlings in micro doses, is a more effective method for providing sufficient nutrition for plant growth and better root development during early stages (**Table 1**). The technology was conceptualized by ICRISAT and it has been repeatedly demonstrated that this approach results in similar or greater yields compared to the broadcasting methods (at recommended dose) while requiring 30-50% less fertilizer (Blessing et al., 2017; Demisie, 2018; Ouedraogo et al., 2020; Ouedraogo et al., 2022) and significantly enhance P nutrition in highly P-fixed soils, and improve P use efficiency (PUE) (Vandamme et al., 2018; Weingartner et al., 2018; De Bauw et al., 2019; De Bauw et al., 2021). Sophisticated agricultural machinery, like seed drill monitoring systems, is used in developed countries to deliver in micro-doses of fertilizer locally to crops (hereafter referred to as MDP), optimizing their use, reducing waste, and minimizing environmental impact. This technology also improves planting accuracy and efficiency, reducing labour costs and promoting sustainable farming practices (Reyes et al., 2015; Simonne et al., 2017; Karimi et al., 2019).

**Table 1:** Comparison of different methods in phosphorus application strategies and efficiency for crop production.

Application Methods		Crop	Soil pH	P source	Application Dose	PUE/PRE	Reference
Broadcasting		Chilli	5.70	SSP	50-200^¶^	≈10% (PUE)	Akinrinde and Adigun (2005)
		Rice	4.31	SSP	60^*^	7% (PRE)	Kalidas-Singh and Thakuria (2018)
		Maize	7.97	DAP	46- 137^*^	26-53% (PUE)	Rafiullah et al. (2020)
		Wheat	5.20	KH_2_PO_4_	30 - 120^¶^	4.2-7.9% (PUE)	Shabnam and Iqbal (2016)
Band application		Maize	7.97	DAP	46- 137^*^	43% (PUE)	Rafiullah et al. (2020)
Localized Application	Micro Dose Placement	Rice	5.7-5.4	TSP	5.5- 11^¶^ or 6.9- 13.7 **^Ψ^**	208%-775% (PRE)	De Bauw et al. (2021)
	Seedling Root Dip in P enriched Slurry	Rice	4.31	SSP	258^¶^ or 2.6**^$^**	155% (PRE)	Kalidas-Singh and Thakuria (2018)
		Onion	8.19	SSP	150^¶^ or 3.5**^$^**	811% (PRE)	Goswami and Kalidas-Singh, (2023)
	Application in Nursery	Cucumber	6.8	MCP	550^¶^	80% (PUE	Liang et al. (2015)
	Fertigation	Maize	7.97	KH_2_PO_4_	144 mM	102% (PUE)	Rafiullah et al. (2020)
Phosphate solubilizing micro-organism with P source		Chilli	6.89	RP	206^¶^	8% (PUE)	Abbasi et al. (2015)
Nano-fertilizers		Peanut	8.20	NZP	164*	33% (PRE)	Hagab et al. (2018)

PUE, P used efficiency; PRE, P Recovery efficiency; SSP, Single Super Phosphate; DAP, Diammonium Phosphate; TSP, Triple Super Phosphate; MCP, Monocalcium phosphate; NZP, Nano Zeolite Phosphorus; ^¶^, Pot experiment (mg P_2_O_5_ kg^-1^); **^Ψ^**, Pot experiment (kg P_2_O_5_ ha^-1^); ^*^, Field experiment (kg P_2_O_5_ ha^-1^); **^$^**, Amount of P_2_O_5_ required in one hectare (2.24 x 10^6^ kg ha^-1^ of soil in 15 cm depth) to make the respective P enriched soil slurry in a 5 m x 9 m (10080 kg of soil in 15 cm depth) corner of the field for dipping seedlings before transplanting in SRD (kg P_2_O_5_ ha^-1^).

In the early stages of growth, crops acquire more P which promotes better root development. When plants reach 25% of their total dry weight, they may have already accumulated about 75% of their total P requirements (Ross and Middleton, 2013). Moreover, research has indicated that the concentration of P in root cells can be up to 1000 times greater than the concentration in the soil solution (Ramaekers et al., 2010). Therefore, it is key to develop a P application method particularly in transplanted crops that facilitates better root architecture during initial crop growth to allow for exploration of more soil volume and enhanced uptake of other essential nutrients. One possible method is to dip seedling roots in P-enriched soil slurry (SRD) just before transplanting, which allows the seedlings to absorb a certain amount of P. Furthermore, P-enriched soil adhered to the roots after dipping acts as a localized P source, enhancing the PUE (Kalidas-Singh and Thakuria, 2018; Goswami and Kalidas-Singh, 2023) (**Table 1**). It is also important to note that excessive exposure to P after certain limit, it can cause toxicity to the plant and reduce P uptake (Oo and Tsujimoto, 2023). Thus, it is crucial to determine the optimal concentration of P and dipping duration. Several studies have reported that the SRD method can increase grain yield of transplanted rice by 10% to 50% with reduced P application rates compared to broadcasting in highly P-fixed acidic soil conditions (Lu and Jiang, 1982; Hooper, 1991; Balasubramanian et al., 1995; Talukdar et al., 2001; Kalidas-Singh and Thakuria, 2018; Oo et al., 2020a; Rakotoarisoa et al., 2020). However, there is no research on this method for other transplanted horticultural crops like Chilli (*Capsicum annuum*) in alkaline soil. Such crops require adequate fertilization, especially with P fertilizers, in order to achieve higher productivity (Sharif and Claassen, 2011; Emongor and Mabe, 2012). Additionally, no study has reported a comparison between the SRD and MDP methods. So, this study aimed in determining the optimal P concentration and incubation duration for SRD method of P application in chilli (*Capsicum annuum*) in terms of biomass production in comparison to the recommended full dose of P by broadcasting method (FD) and micro-dose placement method (MDP).

## 2 Materials and methods

### 2.1 Setup of pot experiment

A pot experiment was set up at the experimental site of College Farm, ITM University, Gwalior, Madhya Pradesh, India, to compare the effectiveness of fertilizer application methods *viz.* Full dose broadcasting method (FD), micro-dose placement method (MDP) and seedling root-dipping into P-enriched slurry methods (SRD). The experiment also aimed to determine the optimum concentration of P and duration of dipping chilli seedlings in P-enriched soil slurry for the SRD method. The objective of the experiment was to identify the best method for achieving optimal results.

**Figure 1:**
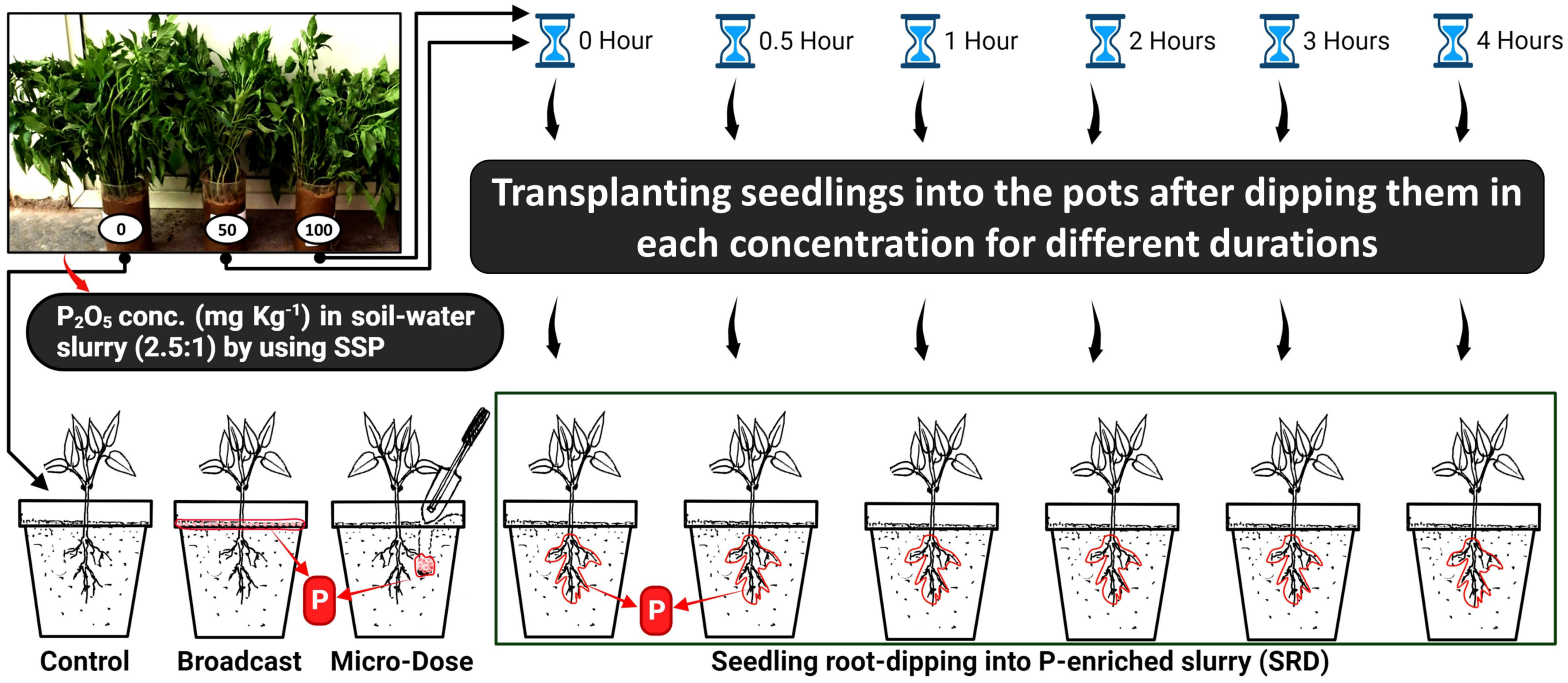
Schematic diagram illustrating the three compared P application methods, including Seedling Root Dip utilizing P-enriched slurry, micro dose placement, and the full dose broadcast approach.

Bulk soils of pH 8.19 (1:2.5; soil:water) were collected from College Farm at a depth of 0-15 cm and mixed thoroughly. The physiochemical properties of the soil are given in **Table 2**. The mixed soils were air-dried, passed through a 2 mm sieve, and used for preparing the slurry for SRD and for conducting the pot experiment. A total of 165 pots (this includes the excluded treatments, explained below), each with a diameter of 30 cm, were filled with 7.0 kg of soil in a manner that ensured the soil’s bulk density remained at 1.25 g cc^-1^. Uniform 40-day-old chili seedlings (variety: *Arka Khyati*) were uprooted from the nursery and the roots were washed under running tap water. The seedling root dip in P-enriched method (SRD method) was prepared according to the method described by Kalidas-Singh and Thakuria (2018). Briefly, 500 g of sieved soil was distributed into plastic beakers (cap. 1000 ml), and 200 ml of water was added to make a soil-water slurry (2.5:1). Five levels of P_2_O_5_ (50, 100, 200, 300, and 400 mg P_2_O_5_ kg^-1^) were imposed by using single superphosphate (SSP; 16% of water soluble P_2_O_5_) which were easily available in market. Another beaker was designated as a control, with no SSP application (0 mg kg^-1^). Bundle of chilli seedling (80 numbers) was dipped in each beaker, and then transplanted to the corresponding pot after a duration of dipping factor (0, ½, 1, 2, 3, and 4 hours). In total, the SRD treatment has 31 treatments including control (0 mg P_2_O_5_ kg^-1^; neglecting dipping duration) which was replicated five times. Two seedlings were transplanted per pot, and four sample per treatment was collected to analyze tissue P concentration (**Figure 1**). The slurry adhered to the seedlings was also collected to determine the amount of P transferred along with the seedlings. [Note: In this experiment, all three levels of P_2_O_5_ (200, 300, and 400 mg kg^-1^), irrespective of the durations of dipping (0, ½, 1, 2, 3, and 4 hours), resulted in death within 7 days after transplantation (refer to **Supplementary Figure S1**). Consequently, these levels were excluded from the subsequent analyses in the manuscript. However, the cause of this mortality is elaborated upon in the discussion section.]

**Table 2:** Soil physicochemical properties in the experimental soil.

Parameters	
Texture	Sandy Clay Loam
**Sand**	67.2%
**Silt**	12.8%
**Clay**	20.0%
**Bulk density (Mg m^-3^)**	1.5 ± 0.1
**Porosity (%)**	48.9 ± 2.3
**Soil pH (1:2.5; soil:water)**	8.2 ± 0.2
**EC (dS m^-1^) (1:5; soil:water)**	3.0 ± 0.1
**OC (%)**	0.4 ± 0.1
**AvlN (kg ha^-1^)**	96 ± 9
**AvlP (kg ha^-1^)**	9.1 ± 0.8
**Exc. K (kg ha^-1^)**	285 ± 20
**CaCO_3_ (%)**	12 ± 0.2
**HCO_3_ (meq 100^-1^ g soil)**	8.48 ± 0.46
**CO_3_ (meq 100^-1^ g soil)**	1.44 ± 0.38
**Exc. Ca (meq 100^-1^ g soil)**	7.66 ± 1.23
**Exc. Mg (meq 100^-1^ g soil)**	1.66 ± 0.32
**Exc. Na (meq 100^-1^ g soil)**	9.5 ± 0.6
**SAR**	6.4 ± 0.6
**Phosphorus Fixation Capacity (%)**	96.2 ± 4.2

The data presented here is obtained from the same experimental soil as utilized in the study conducted by Goswami and Kalidas-Singh, 2023. The soil parameters, including electrical conductivity (1:5), soil pH (1:2.5), soil texture by hydrometer method, bulk density and porosity by Keen box method, organic carbon (OC) by dichromate wet oxidation method, soil available nitrogen (AvlN) by alkaline permanganate method, Available Phosphorus (AvlP) by NaHCO_3_ extractant solution, and exchangeable ((Exc.) potassium by neutral normal ammonium acetate extraction followed by flame photometry, Calcium carbonate, Bicarbonate, Exc. Calcium, Exc. Magnesium, Exc. Sodium and sodium adsorption ratio (SAR) were measured as per standard protocol of Jackson (2005). Phosphorus fixation capacity was analysed by following Dhyan et al. (2005) protocol. In brief, P was added to 2 g of soils in 25μg increments, ranging from 0 to 375μg. After a 96-hour of incubation, it was extracted using Olsen’s reagent and measured AvlP. Finally, P fixation percentage was calculated by subtracting recovered P from the added amount. Values in each parameter are means ± standard errors of 4 replication.

In farmer’s recommended full-dose treatment (FD), P was added by broadcasting (on surface) at a rate of 60 kg P_2_O_5_ ha^-1^ (187.6 mg P_2_O_5_ kg^-1^; this dose is given by Indian Council of Agricultural Research; ICAR in central India) after transplanting. This equated to adding 1170 mg of single superphosphate (SSP) per pot. While, in micro-dose P placement method (MDP), drill placement technique was modified and simulated in the pots as per method described by De Bauw et al. (2021). Specifically, 273 mg of SSP was applied by drilling the topsoil (5 cm deep) near the planting hole of transplanted seedling (2-3 cm). This micro-dose was equivalent to an application rate of 14 kg P_2_O_5_ ha^-1^ when plants are spaced conventionally at 20 cm × 20 cm. Overall, the experiment consisted of a total of 15 treatments with five replications (1 control, 1 FD, 1 MDP and 12 SRD treatment; excluding 200, 300, and 400 mg kg^-1^ P_2_O_5_). In each treatment, including the control, we applied a uniform dose of nitrogen (30 kg N ha^-1^) and potassium (30 kg K_2_O ha^-1^) using urea (0.20 g pot^-1^) and muriate of potash (0.17 g pot^-1^) following farmer’s recommended dosage given by ICAR. All the necessary protective measures and cares were timely employed. Growth and soil properties parameters were measured at 90 days after transplant (DAT).

### 2.2 Soil sampling and chemical analysis

Soil samples were collected from individual pots using a PVC pipe with a 2.54 cm inner diameter. The pipe was inserted to a depth of 10 cm at four locations within each pot. Subsequently, the samples were combined to create one composite sample for each pot and analyzed the levels of available Olsen-Phosphorus (Avl-P) and pH at 90 DAT. The soil pH was measured using a combined glass electrode (Mettler Toledo, Switzerland) method described by Jackson (2005) with soil-to-water ratio of 1:2.5. To determine the Avl-P, 2.25 g of air-dried soil was placed in a 125 ml Erlenmeyer flask, and 50 ml of 0.5N NaHCO_3_ extractant solution (pH 8.5) was added and then filtered through a Whatman No. 40 filter paper (Olsen et al., 1982). The inorganic P concentration in the filtrate was determined using the molybdate blue method at 880 nm wavelength with a spectrophotometer (Murphy and Riley, 1962).

### 2.3 Biomass sampling and chemical analysis

To remove the plants from their pots, the soil was washed in medium-pressure water, and the plants were carefully uprooted. Further, uprooted plants were thoroughly washed in running tap water and then rinsed with distilled water. To remove any excess water, the plants were dried on blotting paper. The root volume of each plant was determined using the volume-displacement technique, in which the roots were suspended in a graduated glass cylinder filled with water (Harrington et al., 1994). The amount of water displaced by the roots was measured using a micropipette with a range of 100 to 1000 µm, allowing for accurate measurement. The whole plant biomass was oven-dried at 65°C to a constant weight and recorded dry biomass production, and ground using a Willey Mill, then stored in an air-tight labelled polyethylene bag.

The determination of P content in plant samples were carried out using the vanadomolybdo-phosphoric yellow colour method (Piper, 1966), wherein 0.5 g of ground plant sample was digested with a di-acid mixture (HNO_3_+HClO_4_) in a 5:2 ratio at a temperature of 250°C for 2.5 h in a digestion block (Kelplus, Pelican Equipment, Chennai). After the addition of the vanadomolybdate reagent, the intensity of the resulting yellow colour was measured using spectrophotometer at a wavelength of 420 nm, and the P concentration was evaluated from the known P standard curve. Finally, the P uptake in the biomass was determined by multiplying the P content (%) with the corresponding dry biomass. P use efficiency (PUE) is the total biomass divided by the fertilizer P applied, while P recovery efficiency (PRE) is derived by subtracting total P uptake from the control Pot (no P input) from that of the fertilized Pot and then dividing by the amount of fertilizer P applied Hussein (2009) and Kumar et al. (2012).

### 2.4 Statistical analysis

The data analysis was done using SPSS v.21. In SRD, 2-factorial ANOVA was performed to determine the main effect of P concentration in the slurry and duration of dipping, as well as their interactions. Tukey’s-b Honestly Significant Difference (HSD) test was used to compare mean values within each factor (concentration and dipping duration). One-way ANOVA was also used to compare with other fertilizer application methods (control, FD and SRD).

## 3 Result

The experimental location exhibits a sub-tropical climate characterized by warm summers and high humidity. Throughout the study period, the average maximum temperature varied between 32°C and 39°C, while the average minimum temperature ranged from 16°C to 21°C. The experimental soil had high soluble salt, low nitrogen and P, but high exchangeable potassium. Its pH was 8.2 (**Table 2**).

### 3.1 Effect of SDR on seedling before transplanting

**Figure 2:**
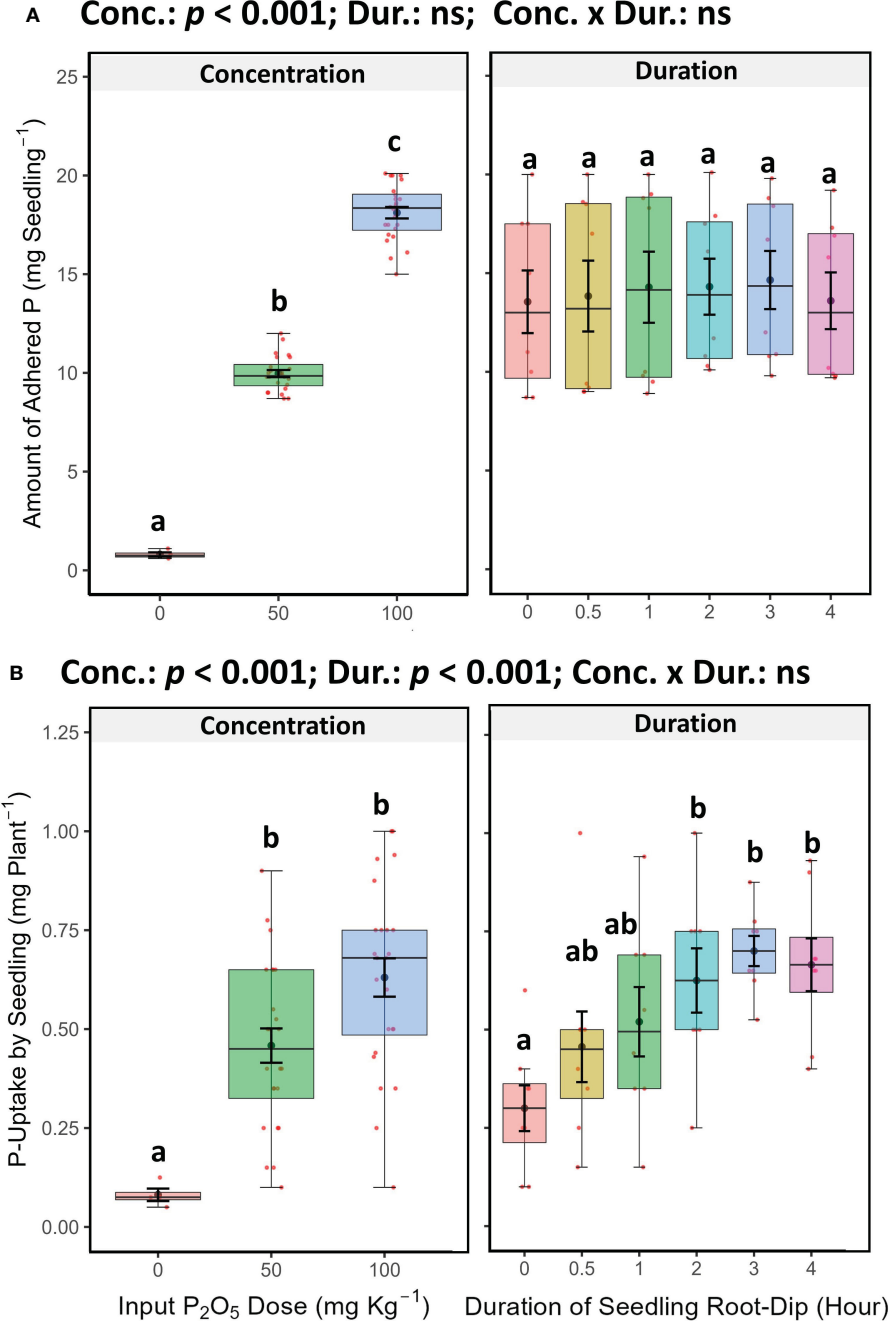
**(A)** Phosphorus (P_2_O_5_) adhesion on roots of Chilli (*Capsicum annuum*) seedlings after dipping in SSP-amended slurries with different P concentrations and duration in SRD method, **(B)** Phosphorus uptake by seedling (variety: *Arka Khyati*; 40 days old) after root dipping in SSP-amended different P concentration slurry and duration. In both cases, mean ± SE of 24 concentration factor measurements and 8 dipping duration factor measurements are shown as thick points with error bars in each box plot. Different letters denote significance at p < 0.05 via Tukey’s HSD, indicating the interaction of concentration and dipping duration in SRD determined by Two-Way ANOVA. “ns” indicates non-significance at 0.05%.

The main effect of P concentration and dipping duration on P uptake in seedlings was statistically significant as determined by two-way ANOVA (*p* < 0.05). Increasing P concentration from 0 to 100 mg P_2_O_5_ kg^-1^ in slurry, enhanced P uptake by seedlings, but no significant difference was observed between 50 and 100 mg P_2_O_5_ kg^-1^ slurry (**Figure 2B**). P uptake was also found to increase with increasing duration of dipping, upto 2 hours, but further exposure did not affect the P uptake. However, the interaction between P concentration and dipping duration (Conc. x Duration) was found to be non-significant (*p* > 0.05; two-way ANOVA).

The soil adhering to the root of the seedling (approximately 0.2 g plant^-1^), were analysed for Avl-P in each treatment. Finally, the total amount of P added in a localized manner (micro-dosing) was calculated for each treatment. Increasing P concentration in the slurry, and the amount of localized P application increased significantly (**Figure 2A**). Compared to other treatments, SRD added much less P per pot (ranging from 13 to 18 mg P_2_O_5_ pot^-1^) than MDP (273 mg P_2_O_5_ pot^-1^) and FD treatments (1170 mg P_2_O_5_ pot^-1^), which were 21 and 90 times higher respectively. However, the duration of dipping didn’t affect the added amount (*p* > 0.05, two-way ANOVA). The interaction between concentration and duration was also non-significant (**Figure 2A**).

### 3.2 Effect of P concentration on slurry and dipping duration in SRD after transplant

Two-way ANOVA showed significant interaction effects between P concentration and dipping duration on biomass, P uptake, PUE, and PRE in SRD (**Tables 3**, **4**). Soil pH had no significant difference among SRD treatments (**Table 3**). Hence, only significant parameters were compared (results below).

**Table 3:** Effect of SRD with different P concentrations and dipping durations on growth of Chilli (*Capsicum Annuum*) growth, compared to control, full dose and micro-dose treatments.

Treatment			Plant Hight (cm plant^-1^)	No of Leaves plant^-1^	Root Volume (cc Plant^-1^)	Dry Biomass (g plant^-1^)	P Uptake (mg plant^-1^)
**Control ^ψ^**			***8.5 ± 1.2^A^***	***14.3 ± 2.1^A^***	***0.6 ± 0.2^A^***	***0.27 ± 0.05^A^***	***0.18 ± 0.03^A^***
**Full Dose**			***11.6 ± 1.4^B^***	***18.0 ± 2.2^AB^***	***0.8 ± 0.2^B^***	***0.41 ± 0.08^AB^***	***0.72 ± 0.15^AB^***
**MDP**			***12.2 ± 1.7^B^***	***17.5 ± 2.6^AB^***	***1.0 ± 0.2^B^***	***0.75 ± 0.04^B^***	***1.70 ± 0.32^B^***
**SRD**			***12.9 ± 1.9^B^***	***20.5 ± 3.1^B^***	***1.9 ± 0.5^C^***	***1.06 ± 0.22^C^***	***2.37 ± 0.68^C^***
Concentration	**50 mg P_2_O_5_ Kg^-1^**	**0 h**	13.2 ± 0.5**^abc^**	19.8 ± 1.0**^abc^**	1.4 ± 0.1**^a^**	0.72 ± 0.04**^a^**	1.70 ± 0.10**^a^**
		**½ h**	12.0 ± 0.7**^abc^**	21.8 ± 1.1**^abc^**	1.5 ± 0.2**^ab^**	0.86 ± 0.02**^ab^**	2.05 ± 0.14**^ab^**
		**1 h**	13.8 ± 1.0**^bc^**	23.3 ± 1.3**^bc^**	1.7 ± 0.1**^abc^**	1.24 ± 0.02**^e^**	2.82 ± 0.15**^b^**
		**2 h**	14.3 ± 0.6**^bc^**	22.9 ± 1.3**^abc^**	2.6 ± 0.1**^ef^**	1.04 ± 0.03**^cd^**	1.96 ± 0.20**^ab^**
		**3 h**	10.9 ± 0.7**^ab^**	22.5 ± 1.3**^abc^**	2.0 ± 0.1**^cd^**	0.88 ± 0.04**^bc^**	1.94 ± 0.07**^ab^**
		**4 h**	10.0 ± 1.0**^a^**	20.0 ± 1.3**^abc^**	1.8 ± 0.1**^abcd^**	0.98 ± 0.04**^bcd^**	2.19 ± 0.25**^ab^**
		***Mean***	***12.4 ± 0.3***	***21.7 ± 0.5***	***1.8 ± 0.1***	***0.95 ± 0.02***	***2.11 ± 0.09***
	100 mg P_2_O_5_ Kg^-1^	**0 h**	13.8 ± 0.8**^bc^**	18.6 ± 1.6**^abc^**	2.0 ± 0.1**^cd^**	1.01 ± 0.05**^bcd^**	2.14 ± 0.15**^ab^**
		**½ h**	13.5 ± 0.7**^bc^**	17.4 ± 1.1**^ab^**	1.5 ± 0.2**^ab^**	1.06 ± 0.04**^d^**	2.60 ± 0.09**^ab^**
		**1 h**	14.7 ± 0.7**^c^**	19.3 ± 1.3**^abc^**	1.8 ± 0.1**^abcd^**	1.27 ± 0.04**^e^**	2.84 ± 0.30**^b^**
		**2 h**	14.2 ± 0.5**^bc^**	23.8 ± 1.1**^c^**	2.9 ± 0.1**^f^**	1.53 ± 0.04**^f^**	3.81 ± 0.35**^c^**
		**3 h**	12.7 ± 0.9**^abc^**	19.3 ± 1.0**^abc^**	2.2 ± 0.1**^de^**	1.08 ± 0.04**^d^**	2.18 ± 0.30**^ab^**
		**4 h**	12.1 ± 0.6**^abc^**	17.1 ± 1.3**^a^**	1.9 ± 0.2**^bcd^**	1.09 ± 0.04**^d^**	2.21 ± 0.31**^ab^**
		***Mean***	***13.5 ± 0.3***	***19.3 ± 0.5***	***2.1 ± 0.1***	***1.17 ± 0.02***	***2.63 ± 0.09***
Dipping Duration	**0 h**		13.5 ± 1.3**^BC^**	19.2 ± 2.6**^A^**	1.7 ± 0.3**^AB^**	0.86 ± 0.17**^A^**	1.92 ± 0.33**^A^**
	**½ h**		12.7 ± 1.5**^ABC^**	19.6 ± 3.1**^A^**	1.5 ± 0.3**^A^**	0.96 ± 0.12**^B^**	2.32 ± 0.37**^AB^**
	**1 h**		14.2 ± 1.6**^C^**	21.3 ± 3.2**^AB^**	1.8 ± 0.2**^AB^**	1.25 ± 0.06**^C^**	2.83 ± 0.44**^B^**
	**2 h**		14.3 ± 1.1**^C^**	23.4 ± 2.2**^B^**	2.8 ± 0.2**^D^**	1.28 ± 0.27**^C^**	2.89 ± 1.12**^B^**
	**3 h**		11.8 ± 1.8**^AB^**	20.9 ± 2.7**^AB^**	2.1 ± 0.1**^C^**	0.98 ± 0.13**^B^**	2.06 ± 0.43**^A^**
	**4 h**		11.1 ± 1.9**^A^**	18.6 ± 2.9**^A^**	1.8 ± 0.3**^BC^**	1.03 ± 0.10**^B^**	2.2 ± 0.53**^A^**
**Conc.**			*	*	*	*	*
**Duration**			*	*	*	*	*
**Conc. x Duration**			**ns**	**ns**	**ns**	*	*

A two-way ANOVA was used to assess the main effects of concentration (n=30) and dipping duration (n=10), as well as their interaction effect. One-way ANOVA was used to compare among SRD groups i.e., within different concentration (mean ± SE; n=5) (lower case parentheses) and duration (mean ± SE; n=10) (upper case parentheses), as well as among full dose (mean ± SD; n=5), MDP (mean ± SD; n=5) and SRD (mean ± SD; n=60) over control (mean ± SD; n=5) (italic bold value and upper-case italic parentheses). Tukey's HSD test was used to determine significant differences between means (p < 0.05). “ψ”, 0 mg P_2_O_5_ Kg^-1^ in SRD; “ns”, non-significant at 0.05% and “*”, significant at 0.05%, “cc” cubic centimetre.

**Table 4:** Effect of SRD with different P concentrations and dipping durations on soil properties, P-use efficiency, and -recovery efficiencies for growing Chilli (*Capsicum Annuum*), in comparison to control, full dose treatments, and micro-dose placement.

Treatment			Soil pH	Soil Avl-P (mg kg^-1^)	P- used Efficiency	P- Recovery Efficiency
**Control ^ψ^**			***8.2 ± 0.1^BC^***	***6.4 ± 0.4^A^***	***NA***	***NA***
**Full Dose**			***8.4 ± 0.1^C^***	***14.0 ± 0.3^c^***	***2 ± 0.1^A^***	***3 ± 1^A^***
**MDP**			***7.9 ± 0.1^AB^***	***10.6 ± 0.5^b^***	***50 ± 3^B^***	***101 ± 21^B^***
**SRD**			***7.6 ± 0.1^A^***	***14.0 ± 0.3^c^***	***123 ± 37^C^***	***252 ± 88^C^***
Concentration	50 mg P_2_O_5_ Kg^-1^	**0 h**	7.7 ± 0.2**^a^**	11.9 ± 0.7**^ab^**	115 ± 6**^cd^**	244 ± 16**^abc^**
		**½ h**	7.5 ± 0.2**^a^**	12.4 ± 0.6**^abc^**	138 ± 3**^e^**	299 ± 22**^bc^**
		**1 h**	7.5 ± 0.1**^a^**	15.0 ± 0.7**^cd^**	198 ± 3**^h^**	423 ± 24**^d^**
		**2 h**	7.7 ± 0.1**^a^**	15.3 ± 0.7**^d^**	166 ± 4**^g^**	285 ± 32**^bc^**
		**3 h**	7.6 ± 0.2**^a^**	14.8 ± 0.6**^cd^**	141 ± 7**^ef^**	282 ± 11**^bc^**
		**4 h**	7.5 ± 0.1**^a^**	14.0 ± 0.4**^bcd^**	156 ± 7**^fg^**	322 ± 41**^c^**
		***Avg.***	***7.6 ± 0.3***	***14.0 ± 0.5***	***152 ± 2***	***309 ± 10***
	100 mg P_2_O_5_ Kg^-1^	**0 h**	7.7 ± 0.2**^a^**	10.8 ± 0.7**^a^**	81 ± 4**^a^**	157 ± 12**^a^**
		**½ h**	7.6 ± 0.1**^a^**	13.9 ± 0.5**^bcd^**	85 ± 3**^ab^**	193 ± 8**^ab^**
		**1 h**	7.5 ± 0.1**^a^**	14.5 ± 0.5**^bcd^**	101 ± 3**^bc^**	213 ± 24**^abc^**
		**2 h**	7.7 ± 0.1**^a^**	15.3 ± 0.4**^d^**	123 ± 3**^de^**	291 ± 28**^bc^**
		**3 h**	7.7 ± 0.1**^a^**	14.8 ± 0.5**^cd^**	86 ± 3**^ab^**	160 ± 24**^a^**
		**4 h**	7.6 ± 0.1**^a^**	15.3 ± 0.5**^d^**	87 ± 4**^ab^**	162 ± 25**^a^**
		***Avg.***	***7.6 ± 0.2***	***14.1 ± 0.5***	***94 ± 2***	***196 ± 10***
Dipping Duration	**0 h**		7.7 ± 0.3**^A^**	11.3 ± 1.4**^A^**	98 ± 21**^A^**	200 ± 53**^A^**
	**½ h**		7.6 ± 0.3**^A^**	13.1 ± 1.3**^B^**	111 ± 29**^B^**	246 ± 64**^AB^**
	**1 h**		7.5 ± 0.2**^A^**	14.8 ± 1.2**^BC^**	150 ± 52**^C^**	318 ± 121**^AB^**
	**2 h**		7.7 ± 0.1**^A^**	15.3 ± 1.0**^C^**	144 ± 24**^C^**	288 ± 56**^AB^**
	**3 h**		7.6 ± 0.3**^A^**	14.8 ± 1.0**^BC^**	114 ± 31**^B^**	221 ± 74**^BC^**
	**4 h**		7.6 ± 0.2**^A^**	14.6 ± 1.1**^BC^**	122 ± 38**^B^**	242 ± 106**^C^**
**Conc.**			**ns**	**ns**	*	*
**Duration**			**ns**	*	*	*
**Conc. x Duration**			**ns**	**ns**	*	*

A two-way ANOVA was used to assess the main effects of concentration (n=30) and dipping duration (n=10), as well as their interaction effect. One-way ANOVA was used to compare among SRD groups i.e., within different concentration (mean ± SE; n=5) (lower case parentheses) and duration (mean ± SE; n=10) (upper case parentheses), as well as among full dose (mean ± SD; n=5), MDP (mean ± SD; n=5) and SRD (mean ± SD; n=60) over control (mean ± SD; n=5) (italic bold value and upper-case italic parentheses). Tukey's HSD test was used to determine significant differences between means (p < 0.05). ^“ψ”^, 0 mg P_2_O_5_ Kg^-1^ in SRD; “ns”, non-significant at 0.05% and “*”, significant at 0.05%, “cc” cubic centimetre.

### 3.3 Effect on plant growth parameters

**Figure 3:**
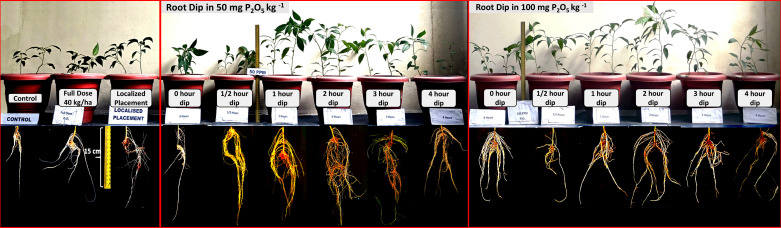
Impact of SRD method on chilli (*Capsicum annuum*) root growth and biomass production at 90 days after transplanting, compared to full dose (60 kg P_2_O_5_ ha^-1^), micro-dose placement (14 kg P_2_O_5_ ha^-1^), and control (no P input) in varied p concentration and dipping durations.

**Figure 4:**
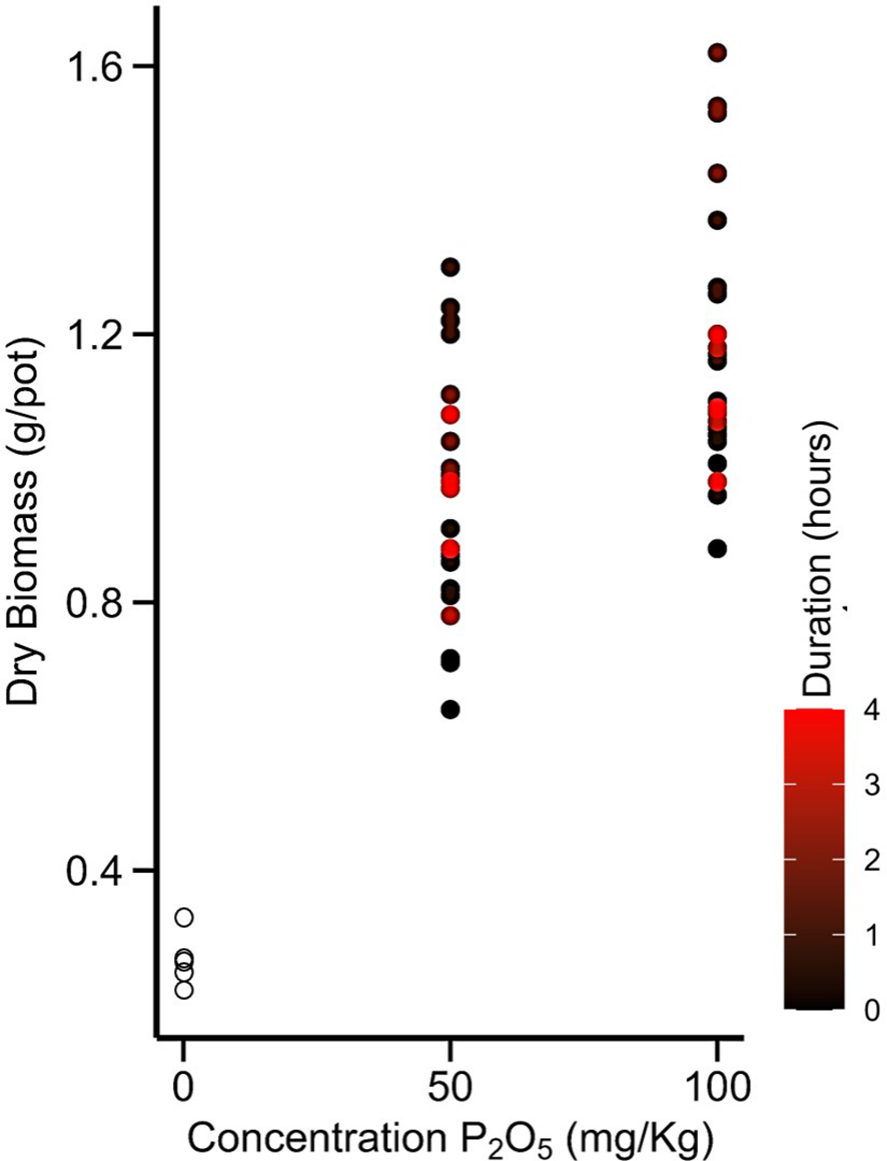
Scatter diagram illustrating the correlation between varying slurry concentrations and the duration of plant dipping, concerning the production of dry biomass at harvest. (Note: The colour scale from black to red indicates seedling dipping duration (in hours) in respective P concentrations, while white circles represent zero dipping duration in 0 mg P_2_O_5_ kg^-1^ concentration).

All the main effect (concentration and duration) of plant growth parameters were found to be statistically significant as determined by two-way ANOVA (*p* < 0.05; **Table 3**; **Figure 3**). In comparison between the mean values of P concentration in slurry, increasing the concentration from 0 to 100 mg P_2_O_5_ kg^-1^ resulted in an increase in plant height, root volume, P uptake, and dry biomass production parameters, except for the number of leaves, which was highest at 50 mg P_2_O_5_ kg^-1^. When considering the dipping duration factor, all growth parameters were highest at 2 hours, but decreased with further exposure to the given concentration (**Table 3**; **Figures 3**, **4**). However, the interaction between concentration and duration showed that only the dry biomass yield and P uptake parameters were statistically significant (*p* < 0.05; two-way ANOVA). Nevertheless, considering the individual factors for their combination (concentration and duration), the highest value was observed in the seedling root dip at 100 mg P_2_O_5_ kg^-1^ for 2 hours, except for plant height, which was highest at 1 hour in the same concentration (*p* < 0.05; one-way ANOVA; **Table 3**; **Figure 3**).

### 3.4 Effect on soil pH and available phosphorus

Soil pH was not affected within SRD treatments. However, with regards to Avl-P, the main effect concentration was statistically non-significant, while the duration factor had a significant impact, as demonstrated by two-way ANOVA (*p* < 0.05; **Table 4**). The highest level of Avl-P was observed after 2 hours of exposure to the slurry. And, the interaction between concentration and duration was not statistically significant. When considering the combinations of concentration with duration of seedling dipping, the highest levels of Avl-P were observed in seeding dipped at 50 mg P_2_O_5_ kg^-1^ for 2 hours followed by 100 mg P_2_O_5_ kg^-1^ for 2 hours, and 100 mg P_2_O_5_ kg^-1^ for 4 hours respectively.

### 3.5 Effect on P-used efficiency and P-recovery efficiency

The main effect of PUE and PRE parameters, namely concentration and duration factors, were statistically significant (two-way ANOVA at *p* < 0.05; **Table 4**). The highest value was observed at 50 mg P_2_O_5_ kg^-1^. When comparing dipping duration, the highest value of PUE was observed in 1 and 2 hours of dipping duration, and the highest value of PRE was observed in 4 hours of dipping duration. The interaction between concentration and duration was statistically significant in both PUE and PRE. Considering the individual treatment, the highest PRE and PUE were observed in 1 hour dipping duration at 50 mg P_2_O_5_ kg^-1^ concentration slurry (one-way ANOVA).

### 3.6 Comparison between the effects of SRD, micro-dose and full dose P application

The mean value of each parameter in SRD methods was compared with those of the control, FD, and MDP, and are presented in **Tables 3**, **4**. Regardless of the application method, the use of P resulted in significant effect in all observed parameters, as determined by one-way ANOVA (*p* < 0.05; **Figure 3**). The details of the differences between FD, MDP, and SRD for each parameter are provided below.

The differences among FD, MDP, and SRD were statistically non-significant for the plant height parameter (*p* > 0.05, one-way ANOVA). However, the number of leaves per plant was significantly higher in the SRD method (*p* < 0.05) than in the FD and MDP methods, although there was no significant difference between FD and MDP. Similarly, the highest root volume was observed in the SRD method of P application compared to the other methods. The percentage increase in root volume from the control was 33%, 67%, and 217% for FD, MDP, and SRD, respectively (**Table 3**; **Figure 4**).

The root volume and plant dry biomass production were found to be highly correlated (correlation coefficient = 0.99). Therefore, the highest dry biomass production was observed in the SRD method of P application compared to the other methods (**Table 3**; **Figure 3**). Additionally, the dry biomass production in the MDP method was higher than in the FD method of P application (*p* < 0.05), as determined by two-way ANOVA (in comparison to the control pot, FD, MDP, and SRD showed increases of 52%, 178%, and 293%, respectively). The P uptake followed the same trend, with the highest value observed in SRD, followed by MDP (*p* < 0.05, one-way ANOVA). Similarly, there was a strong correlation between root volume and P uptake by the plant (correlation coefficient = 0.98). In contrast, Avl-P was not statistically significantly different in FD and SRD treatments, but was significantly higher than MPD (*p* < 0.05), as determined by one-way ANOVA (**Table 4**). Soil pH values were highest in the FD treatment and control, but decreased significantly due to localized P placement (MDP and SRD), with SDR recording the lowest pH value among them. The PUE and PRE were calculated only in FD, MDP, and SRD and were significantly highest in SRD, followed by MDP (*p* < 0.05, one-way ANOVA).

## 4 Discussion

Our study confirms that the application of P through seedling root-dipping into a P-enriched slurry (SRD) for Chilli (*Capsicum annuum*) is more effective than micro-dose placement (MDP) and full-dose broadcast (FD) methods in enhancing biomass production and P uptake in highly P-fixed alkaline soil, while also reducing fertilizer input.

The results showed that increasing the P concentration of the soil slurry (ranging from 0 to 100 mg P_2_O_5_ kg^-1^) led to increase in P uptake by the seedlings before transplanting (**Figure 2A**). A previous study by Shukla et al. (2017) found that P uptake in *Arabidopsis* seedlings grown in hydroponic culture was maximal at 2.5 mM KH_2_PO_4_ solution and further increase in concentration caused a decrease in P uptake. This phenomenon might be attributed to the down-regulation of P uptake transporters such as *PHO1* in the roots under high P concentrations. Similarly, we observed that dipping chilli seedlings in soil slurry with P concentrations of 200, 300, and 400 mg P_2_O_5_ kg^-1^ caused salt stress and death (the data was therefore not included in the statistical analysis; **Supplementary Figure S1**). We also found that the duration of dipping could affect P uptake in seedlings, with no further increase in P uptake observed after 2 hours of exposure period (**Figures 2B**, **4**). This result is consistent with the findings of Kalidas-Singh and Thakuria (2018) in transplanted rice, where a maximum P uptake of 112 mg P_2_O_5_ kg^-1^ was achieved after an 11 hours dip. They suggested that excess P uptake in the seedlings could promote the root proliferation, enable the exploration of additional P in soil, and accelerate post-transplant biomass growth. However, extending the dipping duration caused leaf desiccation shortly and defoliation after transplantation in rice (Oo et al., 2020a). Additionally, the SRD method allowed for localized P fertilization for individual capsicum plant by facilitating seedlings to receive P through slurry adhesion (Ru-Kun et al., 1982; Talukdar et al., 2001) (**Figure 2A**). However, the adhered P-enriched slurry might undergo dilution within the soil after irrigation following transplantation, potentially alleviating any adverse impacts associated with prolonged exposure to higher concentrations. This could explain why the optimal dipping duration, in terms of root volume, number of leaves, biomass production and P uptake, remained consistent at 2 hours. In a recent study on onions, 1-hour dip in slurry with 200 mg of P_2_O_5_ per kg of soil proved most effective (Goswami and Kalidas-Singh, 2023). Both onions and chilli are horticultural crops transplanted into aerated soil. Whereas, when rice is in flooded soil, it dilutes the adhered P-enriched slurry, possibly extending the optimum dip to 11 hours (Kalidas-Singh and Thakuria, 2018). In chilli, it’s worth mentioning that the P-enriched slurry adhered to the seedling roots remained highest in amount at 100 mg P_2_O_5_ kg^-1^ compared to 50 mg P_2_O_5_ kg^-1^, and acted as a localized and continuous source of P and creates soluble P hot-spots around the root zone (Ru-Kun et al., 1982; Oo et al., 2020b) resulting in increased root proliferation and greater soil volume contact after transplanting (**Table 3**). This ultimately facilitates greater uptake of all essential nutrients in the future. However, the duration of dipping into high P concentration slurry also has a significant post-transplant impact, as prolonged exposure to high P concentrations during SRD might cause root injury and reduction in root volume. Previous studies have also highlighted this phenomenon in rice (Ru-Kun et al., 1982; Shukla et al., 2017; Oo et al., 2020b). Our findings, suggest that dipping chilli seedlings in a slurry with high levels of P reduced the roots proliferation, but shorter dipping durations can help reduce the negative effect to the roots (**Table 3**; **Figure 4**). And root volume also positively impacts on Avl-P. Roots releases several enzymes, typical organic acids like malic, malonic, acetic, citric, fumaric, succinic, lactic, tartaric, oxalic, and others during root exudation, which has P-solubilizing capabilities that converts unavailable P into bio-available P. Generally, these very similar enzymes and organic acids are also produced by rhizospheric microbes (Koo et al., 2005; Chen et al., 2008), which could potentially be more abundant in larger root volumes. This may result in the highest Avl-P after 2 hours of dipping in both concentrations with maximum root volume. So, root volume plays a crucial role in the SRD. P fixation might be more prominent in soils with higher application rates, such as FD, compared to others. Since the soil has a higher P fixation rate (**Table 2**), soluble P may react with exchangeable calcium or magnesium, leading to the formation of insoluble P (P-fixation) and potentially causing an increase in soil pH. However, the small amount of P supply through SRD had no significant effect on soil pH among the SRD treatment, resulting in high PUE or PRE and reduced P fixation (Oo et al., 2020b; Kalidas-Singh et al., 2021; Goswami and Kalidas-Singh, 2023). So, to prevent harmful consequences of extended dipping periods in high P concentration slurry on SRD, it is advisable to immerse seedlings in a P-enriched slurry containing 100 mg P_2_O_5_ kg^-1^ for no longer than 2 hours during chilli cultivation in alkaline soil (pH 8.2).

When comparing the effects of P applications (SRD, MDP, and FD) at 90 DAT, it was evident that the MDP and SRD, both emphasizing localized P placement principles, outperformed the FD broadcast approach across nearly all parameters. Localized P placement promotes root proliferation, expanding the root absorption surface area and capturing more soil nutrients, as supported by previous studies (Jing et al., 2010; De Bauw et al., 2021). This can explain the increase in dry biomass production and P uptake in our finding (**Table 3**), as root proliferation in nutrient-rich areas can compensate for uneven nutrient distribution across the root system (Robinson, 2001).

However, the type of localized P placement, specifically between SRD and MDP, can also affect root proliferation. Several studies have shown that fertilizer P placement and distance from the plant have a significant impact on crop yield, nutrient uptake, and fertilizer usage efficiency due to P’s immobility in soil. Closer placement of fertilizer has been observed to improved biomass yield (Sander and Eghball, 1999; Hu et al., 2018). In our study, the SRD approach resulted in greater root proliferation than the MDP approach due to its closer proximity to the plant (**Figure 1**). Additionally, dipping the seedlings in P enriched slurry before transplanting in the SRD resulted in extra P uptake on seedlings compared to the MDP approach (0 mg P_2_O_5_ kg^-1^ is similar to the seedlings of MDP; **Figure 2B**). So due to all these factors, the SRD approach has been observed to contribute better biomass yield than MDP and FD (Kalidas-Singh and Thakuria, 2018).

Although, FD treatment (added P amount:170 mg P_2_O_5_ pot^-1^) obtained 90 times more P than SRD treatment (added P amount:13 to 18 mg P_2_O_5_ pot^-1^), the influence of soil Avl-P was not significant for both the treatments (**Table 4**). As previously explained, a substantial amount of native soil P in SRD treatment may have been converted into bio-available P due to the release of phosphatase enzymes during root exudation and microbial activities, which might be more active in SRD due to its higher root volume (Chen et al., 2008). Despite the observed phenomenon, MDP treatment did not experience the similar impact as compared with SRD and FD, which is consistent with the findings of Oo et al. (2020b) in their study on rice.

The FD treatment resulted in high soil pH, but its low PUE and PRE values indicated a significant tendency of P fixation in the soil (initial P fixation capacity of the soil was 96%, **Table 2**). However, localized placement of P using MDP or SRD can decrease soil pH due to increased root proliferation, which releases organic acids such as citric, malic, and oxalic, leading to lower soil pH (Bloom et al., 2002; Jing et al., 2010). This can have a negative effect on soil calcification and ultimately enhances P availability and uptake (Wang et al., 2018). The PUE and PRE values for the MDP treatment were consistent with the findings of previous studies (He et al., 2003; Jing et al., 2010; Simonne et al., 2017), while those for the SRD treatment were much higher, suggesting that the crops may have utilized native P from the soil (Kalidas-Singh and Thakuria, 2018). However, to maintain balanced P pool in highly P-fixed soils, it is very necessary to supplement the SRD treatment with other P sources (like Rock phosphate, organic matter, etc) in reduced doses. Since P is an essential macro element, but in the practical field approach, the supplied P in SRD treatment will be very small quantity. For example, the finding, 100 mg P_2_O_5_ kg^-1^ slurry and two-hour dipping period (which results in the highest biomass production) can be achieved by applying 9.5 kg of SSP to a 5m x 9m area (10,080 kg soil in 15cm depth) in the corner of a one-hectare area and dipping the seedlings for 2 hours before transplanting them with a spacing of 20cm x 20cm. So, the total P needed is approximately 1.51 kg P_2_O_5_ ha^-1^.

## 5 Conclusions

The Seedling Root-Dip (SRD) technique, in which seedlings are dipped in a P-enriched slurry before transplant, is an innovative method of P application in transplanted crops like chilli. The best outcome (in term of biomass production) was achieved with a soil slurry of 100 mg P_2_O_5_ kg^-1^ for 2 hours dipping duration. It was also observed that high P concentration in slurry and longer dipping durations had negative effects on plant and root growth. Compared to the micro-dose placement and full dose broadcasting methods, the SRD treatment showed improvement in root proliferation, biomass production, as well as more efficient P utilization and recovery. To maintain balanced P retention in highly P-fixed soil, it is recommended to use other P sources in reduced doses along with the SRD technique. Overall, from this study, the potential of the SRD technique for improving P management in agriculture, particularly for transplanted crops, can be promulgated.

## Data availability statement

The original contributions presented in the study are included in the article/**Supplementary Material**, further inquiries can be directed to the corresponding author.

## Author contributions

SS: Funding acquisition, Investigation, Writing – original draft. SK-S: Conceptualization, Data curation, Formal analysis, Investigation, Methodology, Project administration, Resources, Software, Supervision, Validation, Visualization, Writing – original draft, Writing – review & editing. AP: Supervision, Writing – original draft, Writing – review & editing. DT: Conceptualization, Data curation, Writing – review & editing. SM: Writing – review & editing. RZ: Writing – review & editing. AD: Writing – review & editing. SB: Writing – review & editing. MH-D: Conceptualization, Investigation, Writing – review & editing. SH: Writing – review & editing. TT: Writing – review & editing. YU: Visualization, Writing – review & editing. PB: Formal Analysis, Writing – review & editing. VR: Visualization, Writing – review & editing. BL: Writing – review & editing. AT: Writing – review & editing. DS: Writing – review & editing.

## Supplementary material

Supplementary Figure 1 Plants exposed to higher concentrations of P, particularly at or above 200 mg P_2_O_5_ kg^-1^, exhibited mortality after being dipped for 0 hours.
