# Supplementary figures and images for: A comparative study of localized phosphorus application and broadcasting method on biomass production and their use efficiency on Chilli (Capsicum annuum) under alkaline soil
Source: Front Plant Sci. 2024 Mar 1;15:1304963. doi: 10.3389/fpls.2024.1304963 (PMC10940543; doi:10.3389/fpls.2024.1304963)

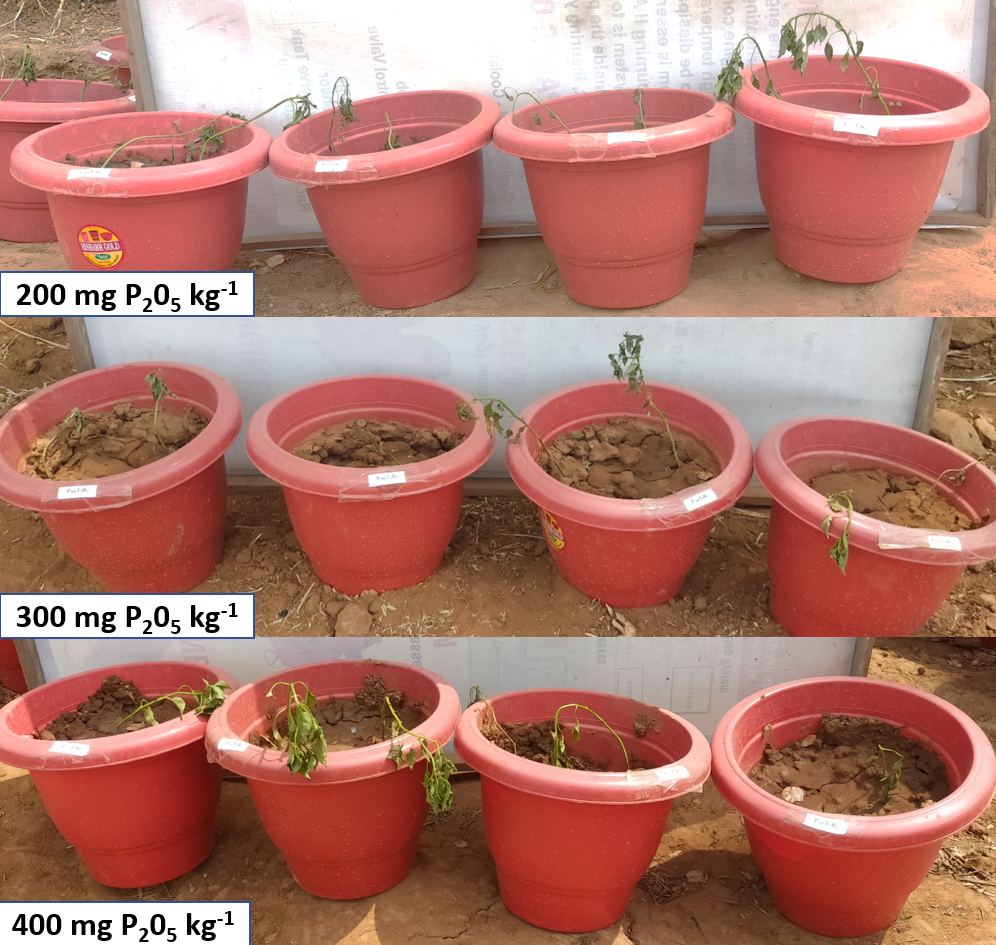

Supplement: Supplementary Figure 1 — Plants exposed to higher concentrations of P, particularly at or above 200 mg P2O5 kg-1, exhibited mortality after being dipped for 0 hours. [file Image_1.jpeg]
